# (±)-Pestalachloride D, an Antibacterial Racemate of Chlorinated Benzophenone Derivative from a Soft Coral-Derived Fungus *Pestalotiopsis* sp

**DOI:** 10.3390/md11041050

**Published:** 2013-03-28

**Authors:** Mei-Yan Wei, Dan Li, Chang-Lun Shao, Dong-Sheng Deng, Chang-Yun Wang

**Affiliations:** 1 Key Laboratory of Marine Drugs, Ministry of Education, School of Medicine and Pharmacy, Ocean University of China, Qingdao 266003, China; E-Mails: mywei95@126.com (M.-Y.W.); lidaneasy@163.com (D.L.); 2 School of Pharmacy, Guangdong Medical College, Dongguan 523808, China; 3 College of Chemistry and Chemical Engineering, Luoyang Normal University, Luoyang 471022, China; E-Mail: dengdongsheng168@sina.com

**Keywords:** (±)-pestalachloride D, antibacterial activity, zebrafish embryo teratogenicity assay, marine-derived fungus, *Pestalotiopsis* sp., single-crystal X-ray

## Abstract

A new antibacterial chlorinated benzophenone derivative, (±)-pestalachloride D (**1**), along with a related analog, (±)-pestalachloride C (**2**), was recently isolated from the marine-derived fungus *Pestalotiopsis* sp. isolated from a soft coral *Sarcophyton* sp. collected from Yongxing Island in the South China Sea. Both chiral HPLC analysis and single-crystal X-ray data indicated that **1** is a racemic mixture. Interestingly, **1** did not exhibit any effect in the zebrafish embryo teratogenicity assay, while **2** led to abnormal growth. The potential impact on zebrafish embryo growth is discussed based on their crystal structures. The main difference of crystal structures between **1** and **2** is that the six-member non-aromatic ring (O4, C10, C9, C8, C2′, and C3′) in **1** exhibits a distorted chair conformation, while **2** shows a distorted boat conformation. Moreover, compounds **1** and **2** both exhibited moderate antibacterial activity.

## 1. Introduction

Over the past two decades, marine-derived fungi have proven to be rich sources of structurally novel and biologically active secondary metabolites, which have become significant resources for drug discovery [1,2]. Furthermore, a growing body of evidence indicates that the genus *Pestalotiopsis* represents a huge and largely untapped resource of natural products with chemical structures that have been optimized by evolution for biological and ecological importance [3]. During the past two decades, about 200 fungal secondary metabolites including terpenoids, alkaloids, lactones, and coumarins, have been reported from this genus [3]. 

**Figure 1:**
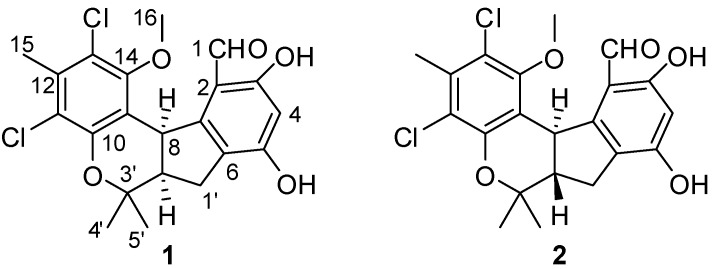
Structures of compounds **1** and **2**.

Chemically-induced teratogenicity is preventable with proactive reproductive safety evaluations. The developing zebrafish (*Danio rerio*) is an *in vivo* developmental model with a number of advantages over *in vitro* systems and provides a simple, inexpensive and rapid assay to screen for teratogenicity [4,5]. As part of our ongoing investigation into new natural metabolites, a series of antibacterial, antifouling and cytotoxic products have been isolated from marine fungi from the South China Sea [6,7,8,9]. Under the guidance of zebrafish embryo teratogenicity assay, a new chlorinated benzophenone derivative, (±)-pestalachloride D (**1**), along with one related known analogue, (±)-pestalachloride C (**2**) [10] (Figure 1), was obtained from the organic extracts of the marine-derived fungus *Pestalotiopsis* sp. The fungus was isolated from a soft coral *Sarcophyton* sp. collected from Yongxing Island in the South China Sea. Both **1** and **2** are dichlorinated and were found to be racemic mixtures by chiral HPLC analysis and single-crystal X-ray data. 

Herein, we report the isolation, structure determination, chiral HPLC analysis, antibacterial activity and zebrafish embryo teratogenicity effect for the racemic mixtures. The potential impact on zebrafish embryo growth is also discussed based on the crystal structures.

## 2. Results and Discussion

The marine-derived fungus ZJ-2009-7-6, isolated from the soft coral *Sarcophyton* sp., was identified by molecular characteristics along with morphologic traits as a *Pestalotiopsis* sp. Both (±)-pestalachloride D (**1**) and (±)-pestalachloride C (**2**) were isolated using chromatographic techniques, including column chromatography and semi-preparative HPLC, and their structures were elucidated by comprehensive spectroscopic data (IR, UV and NMR), along with HREIMS and X-ray diffraction analyses. 

**Figure 2:**
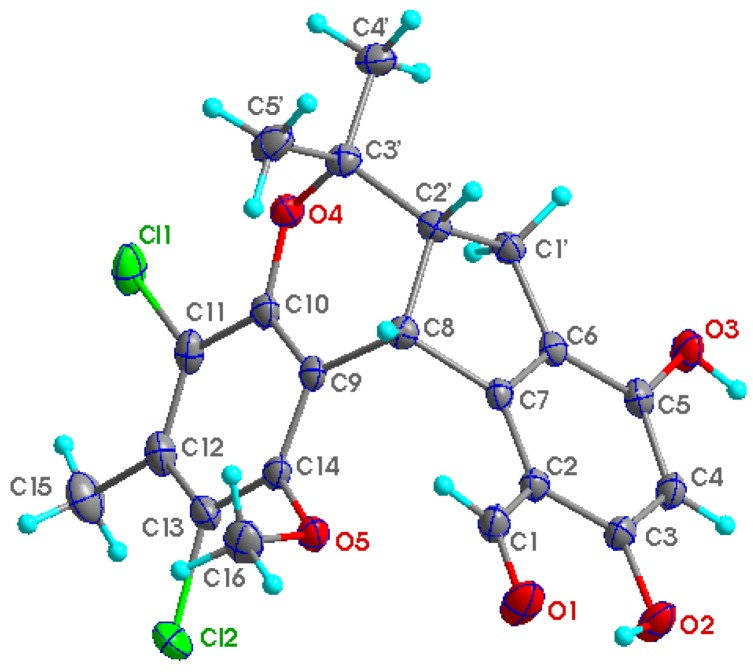
Perspective ORTEP drawing for **1**.

Compound **1** was obtained as colorless crystals. The HREIMS data (*m/z* 422.0678, calcd 422.0682) together with its ^13^C NMR spectrum indicated that it has a molecular formula of C_21_H_20_Cl_2_O_5_ with 11 degrees of unsaturation. Furthermore, the ratio of [M]^•+^ isotopic peaks (422:424:426/10:6:1) clearly indicated the presence of two chlorine atoms. The ^13^C NMR and DEPT spectra revealed 21 carbon signals, including one appearing to be an aldehyde group (δ_C_ 193.4) and twelve aromatic carbons, representing two phenyl rings. With nine of the 11 degrees of unsaturation accounted for, the structure of **1** was suggested to contain another two rings. The IR absorption bands at 3444 and 1647 cm^−1^ suggested the presence of both a hydroxyl and carbonyl groups. Careful comparison of the ^1^H NMR and ^13^C NMR data of **1** (Table 1) with those of (±)-pestalachloride C (**2**), which was previously reported from the plant endophytic fungus *Pestalotiopsis adusta* (L416) [10], showed close structural homology to **2**. The most obvious differences involved coupling constants. In **1**, a small coupling constant (*J* = 6.0 Hz) was observed between H-8 (δ_H_ 4.71) and H-2′ (δ_H_ 2.81), instead of a large coupling constant observed (*J* = 11.2 Hz) between H-8 (δ_H_ 4.26) and H-2′ (δ_H_ 2.30) in **2**. This small coupling constant between H-8 and H-2′ indicated a *cis*-relationship between these protons, where as in **2** it has been reported to have a *trans*-related. Finally, by slow crystallization from MeOH, single crystals of **1** suitable for X-ray diffraction analysis using Cu Kα radiation were obtained, allowing the structure of **1** to be unambiguously established with H-8 and H-2′ in a *cis* relationship (Figure 2). Single crystals of **2** were also obtained and measured. According to the crystal data of **1** and **2**, the dihedral angles between H-8 and H-2′ were calculated to be *ca.* 35° and 159°, respectively, which were consistent with the values deduced by the Karplus formula. The planar and relative structure of **1** was completely determined as shown in Figure 2. 

**Table 1:** ^1^H and ^13^C NMR Data for **1** ^a^.

Position	1	
	δ_C_	δ_H_ (*J*/Hz)
1	193.4	10.21 (1H, s)
2	112.9	
3	163.6	
4	102.1	6.20 (1H, s)
5	158.5	
6	120.0	
7	148.5	
8	38.7	4.71 (1H, d, 6.0)
9	121.4	
10	150.0	
11	114.0	
12	135.6	
13	119.4	
14	154.0	
15	17.9	2.42 (3H, s)
16	60.2	3.59 (3H, s)
1′	27.5	2.81 (2H, overlapped)
2′	51.1	2.81 (1H, overlapped)
3′	75.6	
4′	26.8	1.55 (3H, s)
5′	24.6	1.38 (3H, s)
OH-3		11.74, brs

^a^ Measured at 400 MHz (^1^H) and 100 MHz (^13^C), CDCl_3_.

The space group *P*2_1_/c of the crystallographic data indicated **1** is a racemate with *SS*/*RR* configurations. The specific rotation [α]^25^_D_ 0 (*c* 1.00, CHCl_3_) and the absence of a CD maximum also confirmed the above result. This was further supported by subsequent HPLC analysis of (±)-**1** on a chiral phase column, in which two distinct chromatographic peaks appeared with a ratio of 1:1. In fact, several natural products including (±)-pestalachloride C [10], sporothrins A and B [11], and longamide [12] have also been found as racemic mixtures.

**Scheme 1:**
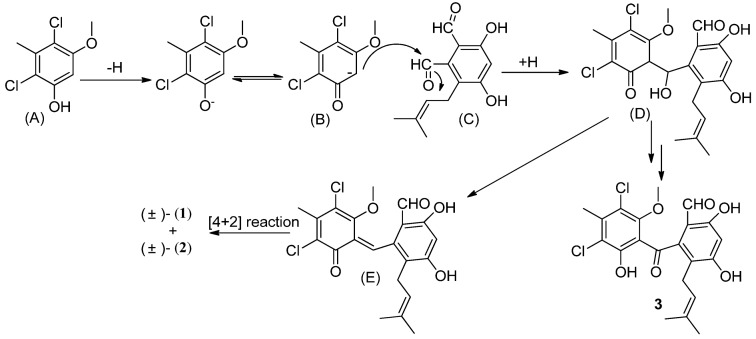
Possible biogenetic path of (±)-pestalachlorides D (**1**) and C (**2**).

A possible biogenetic pathway of (±)-pestalachlorides D (**1**) and C (**2**) is proposed as shown in Scheme 1. The dichloro-substituted phenol (A) is firstly deprotonated, and resonanized to a stabilized carbanion (B). Then the resulting carbanion undergoes an Aldol reaction by attacking the carbonyl group of the aldehyde (C) to generate a tetrahedral intermediate followed by capturing a proton to form a secondary alcohol (D). The resulting alcohol dehydrates to an α,β-unsaturated ketone (E), then undergoes an intramolecular [4 + 2] hetero-Diels–Alder reaction to afford the product, (±)-pestalachloride D (**1**) and (±)-pestalachloride C (**2**). This deduction was supported by the co-isolation of the intermediate metabolite, pestalone (**3**), which was isolated from the plant endophytic fungus *Pestalotiopsis adusta* (L416) [10]. Interestingly, a novel meroterpenoid, (±)-Guajadial B, was recently isolated from *Psidium guajava* and also synthesized by biomimetic strategy which also contains a hetero-Diels Alder reaction [13].

All the biological activities of compounds **1** and **2** were evaluated as their racemic forms. Compounds **1** and **2** were evaluated in the zebrafish (*D. rerio*) embryo teratogenicity assay. To our surprise, compound **1** was inactive at a concentration of 50 μg/mL, while **2** led to abnormal growth effects in several aspects of the embryonic development. For example, **2** exhibited coagulated eggs (24 h), non-spontaneous movements (24 h), abnormal heartbeat (48 h), tail malformation (48 h), heart malformation (48 h), notochord malformation (72 h), delayed hatch (72 h) and embryo death (72 h) with EC_50_ values of 16.3, 18.6, 6.3, 24.5, 8.2, 5.8, 7.4 and 12.6 μg/mL, respectively. These results indicate that the *trans* relative configuration between H-8 and H-2′ in **2** potentially contributes to the toxic effects of **2**. 

**Figure 3:**
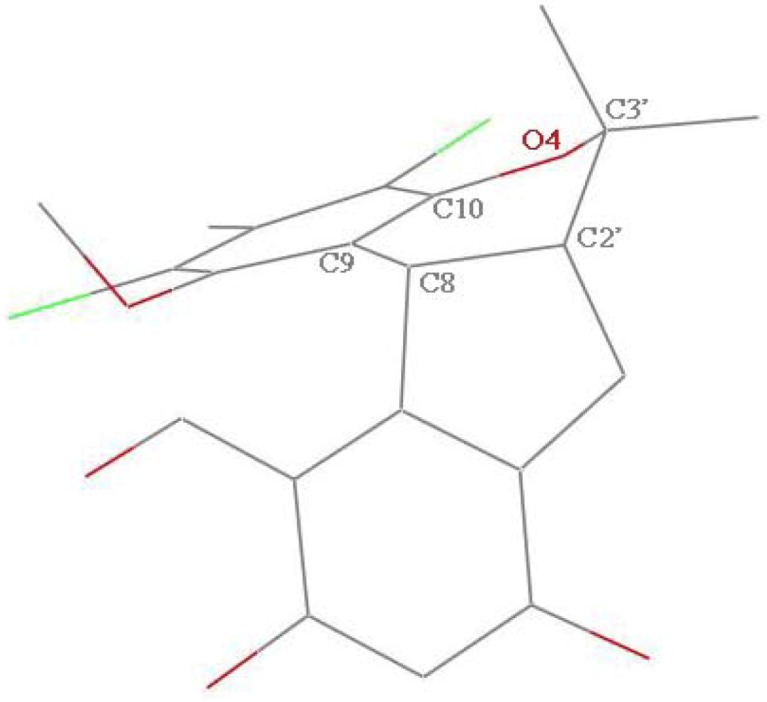
The distorted half-chair conformation of six-membered non-aromatic ring (C8, C9, C10, O4, C3′, C2′) in **1**.

**Figure 4:**
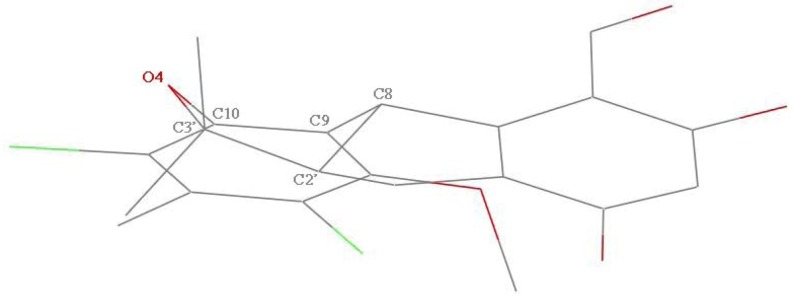
The distorted boat conformation of six-membered non-aromatic ring (C8, C9, C10, O4, C3′, C2′) in **2**.

**Figure 5:**
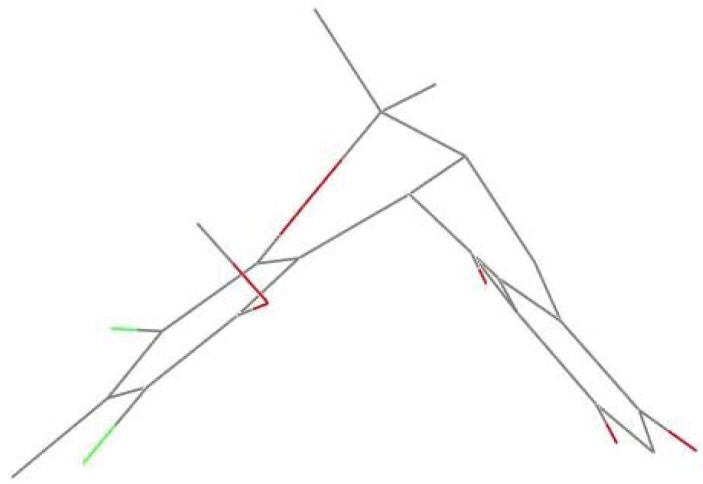
The “butterfly” conformation of **1**.

**Figure 6:**
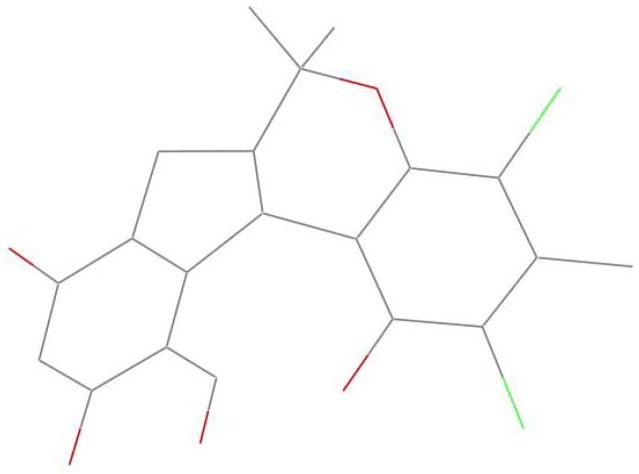
The “butterfly” conformation of **2**.

In order to explain the possible conformational differences and their effects on toxicity, we focus on the two crystal structures. Here are some obvious conformational differences of two non-benzene rings. Firstly, the crystal of compound **1** is monoclinic system with space group *P*2_1_/c, and crystal of **2** is triclinic system in space group *P*ī. Secondly, in the crystal of **1**, one six-member non-aromatic ring (O4, C10, C9, C8, C2′, and C3′) exhibits a distorted chair conformation with atoms O4 and C8 deviating by 0.405(4) and −0.352(4) Å, respectively, from the mean plane of the other four atoms (Figure 3). Correspondingly, in the crystal of **2**, the non-aromatic six-membered ring showing a distorted boat conformation is *anti*-fused along the C8–C2′ bond to a five-membered ring, placing the atoms O4 and C8 on the prow and stern positions deviating by 0.376(3) and 0.778(3) Å, respectively, from the mean plane of the other four atoms (Figure 4). Thirdly, in compound **1**, the five-membered ring (C7, C6, C1′, C2′ and C8) with a *cis*-junction to the six-membered non-aromatic ring along the C8–C2′ bond adopts a envelope conformation with C2′ away from the mean plane of the other four atoms by −0.489(3) Å. It should be noted that the tetracyclic skeleton of **1** exists predominantly in a “butterfly” conformation, in which the dihedral angle between the two wings formed by two aromatic rings is about 82.8(5)°. This arrangement makes the methoxy group tilt out of the corresponding aromatic ring (dichlorophene) by 79.5°, appearing on the α-face of the molecule (Figure 5). However, in compound **2**, the five-membered ring (C6, C7, C8, C2′, and C1′) displays an envelope conformation with C2′ deviating out of the mean plane of the other four atoms by −0.427(3) Å. It must be pointed out that the structural landscape of **2** exhibits a “butterfly” conformation, in which two wings constructed by two aromatic rings form the dihedral angles of 51.4(3)°. Furthermore, the methoxy group deviates out of the dichlorophene about 89.9°. Within this mode, the methoxy group and methyl group (C5′) are projected on β-face of the molecule (Figure 6). These above configurational differences could partly explain why the two compounds have so obviously different effects on the embryonic development of the zebrafish. Further studies on the effect mechanisms are in progress.

Compounds **1** and **2** both exhibited moderate antibacterial activity against *Escherichia coli*, *Vibrio anguillarum* and *Vibrio parahaemolyticus* with the MIC values of 5.0, 10.0 and 20.0 μM, respectively. Neither of them showed any inhibitory activity against *Micrococcus tetragenus* at a concentration of 20.0 μM.

Moreover, **1** and **2** did not show any herbicidal activity against *Arabidopsis thaliana* at 10 ppm and *Poa annua* at 32 ppm. Nor did they exhibit any insecticidal activity against *Plutella xylostella* and *Diabrotica balteata* at 500 ppm in artificial diet assays, and the mycelial growth tests in artificial media against *Pythium dissimile*, *Alternaria solani*, *Botryotinia fuckeliana* and *Gibberella zeae* at rates of 20 and 2 ppm.

## 3. Experimental Section

### 3.1. General Experimental Procedures

Melting points were determined on an X-6 micromelting point apparatus and are uncorrected. Optical rotations were measured on a JASCO P-1020 digital polarimeter. UV spectra were recorded on an UV-2501PC spectrophotometer. IR spectra were recorded on a Bruker EQUINOX 55 spectrometer using KBr pellets. NMR spectra were recorded on a JEOL JEM-ECP NMR spectrometer (600 MHz for ^1^H and 150 MHz for ^13^C) and on a Bruker AVANCE 400 NMR spectrometer (400 MHz for ^1^H and 100 MHz for ^13^C). Chemical shifts δ are reported in ppm, using TMS as internal standard and coupling constants (*J*) are in Hz. EIMS spectra were measured on a Thermo DSQ EI-mass spectrometer and HREIMS on a Thermo MAT95XP High Resolution mass spectrometer. Single-crystal data was measured on an Agilent Gemini ultra diffractometer (Cu Kα radiation). HPLC separation was performed in a Waters 1525 preparative HPLC system coupled with Waters 2996 photodiode array detector. A Kromasil C_18_ preparative HPLC column (250 mm × 10 mm, 5 μm) and a chiral Crownpak CR (+) column (150 mm × 4 mm, 5 μm) were used. Silica gel (Qing Dao Hai Yang Chemical Group Co.; 200–300 mesh), octadecylsilyl silica gel (Unicorn; 45–60 μm) and Sephadex LH-20 (GE Healthcare, Bio-Science AB, Sweden) were used for column chromatography (CC). Precoated silica gel plates (Yan Tai Zi Fu Chemical Group Co.; G60, F-254) were used for thin layer chromatography (TLC). The antibacterial activities were observed with a Multiskan Mk3 (Thermo Labsystems) at 630 nm. A vertical heating pressure steam sterilizer LDZX-75KBS was used for the sterilization of culture medium. The fungi were cultivated in a Biochemical Incubator (Model SPX-250B-Z) or SHIPING Constant Temperature Culture Vibrator at 25 °C with a speed of 160 rpm. 

### 3.2. Isolation of the Fungal Material

The fungal strain *Pestalotiopsis* sp. (ZJ-2009-7-6) was isolated from a piece of fresh tissue from the inner part of a soft coral *Sarcophyton* sp., collected from Yongxing Island in the South China Sea in November, 2009. The strain was deposited at the Key Laboratory of Marine Drugs, the Ministry of Education of China, School of Medicine and Pharmacy, Ocean University of China, Qingdao, China. Following surface sterilization with 75% EtOH for 30 s, the soft coral was rinsed three times in sterile water. To distinguish the remaining epiphytic fungi from endophytic fungi, an imprint of the soft coral surface on PDA was made. Small tissue samples from inner part of the soft coral were aseptically cut and pressed onto PDA plates containing an antibiotic to suppress bacterial growth (composition of isolation medium: 200 g/L potatoes, glucose 20 g/L, agar 15 g/L, and chloramphenicol 0.2 g/L in sea water, pH 7.4–7.8). After incubation at 25 °C, the fungal strain under investigation was found to grow exclusively out of the soft coral tissue, but not on the agar plates taken from the imprint of the soft coral surface. A pure strain of *Pestalotiopsis* sp. (ZJ-2009-7-6) was isolated from the growing cultures by repeated re-inoculation on PDA plates. 

### 3.3. Identification of Fungal Cultures

The fungus was identified as *Pestalotiopsis* sp. according to morphologic traits and a molecular biological protocol by DNA amplification and sequencing as described below. About 100 mg of fresh fungal mycelium was collected in a microcentrifuge tube (1.5 mL) to extract genomic DNA from the fungus using the Fungal DNA Kit (50) (E.Z.N.A., Omega) according to the manufacturer’s protocol. The PCR reactions were performed in a final volume of 50 μL which was composed of template DNA (2 μL), 5 μL 10× buffer, 1 μL dNTP, 0.5 μL ITS1F, 0.5 μL ITS4 (20 μmol/mL each), 0.25 μL of Taq polymerase and appropriate ultrapure water under the following conditions: (1) initial denaturation at 94 °C for 5 min; (2) denaturation at 94 °C for 40 s; (3) annealing at 52 °C for 40 s; (4) extension at 72 °C for 1 min; (5) final extension at 72 °C for 10 min. Steps 2–4 were repeated 30 times. Then, 5 μL of the amplification products was loaded onto an agarose gel (1.2% agarose in 0.5× TAE, 5 μL of ethidium bromide 1% m/v solution per 100 mL of gel). After electrophoresis at 100 V for 35 min, the band due to the PCR product (approximate size 526 bp) was isolated from the gel slice using the Gel Extraction Kit (E.Z.N.A., Omega) according to the manufacturer’s protocol. The PCR product was then submitted for sequencing (Invitrogen, Shanghai) with the primer ITS4. The fungus was identified as a *Pestalotiopsis* sp. whose 617 base pair ITS sequence had 99% sequence identity to that of *Pestalotiopsis* sp. DFFW (EF055190). The sequence data have been submitted to GenBank, accession number HM486429. 

### 3.4.Fermentation, Extraction and Isolation

The fungal strain *Pestalotiopsis* sp. (ZJ-2009-7-6) was cultivated in 20 L liquid medium (composition of medium: 200 g/L cooked and sliced potatoes, 20 g/L glucose in artificial sea water, in 1 L Erlenmeyer flasks each containing 400 mL of culture broth) at 27 °C without shaking for 4 weeks. The culture was filtered to separate the culture broth from the mycelia. Then the fungal mycelia were extracted three times with EtOAc and three times with CHCl_3_/MeOH (v/v, 1:1). The organic extracts were combined and concentrated under vacuum to afford a dry crude extract (6.2 g). The resulting exact (6.2 g) was subjected to silica gel column chromatography (CC) (petroleum ether, EtOAc v/v, gradient elution) to offer seven fractions (Fr. 1–Fr. 7). Fr. 3 was subjected to silica gel CC with petroleum ether-EtOAc (4:1) and Sepphadex LH-20 CC eluting with mixtures of petroleum ether-CHCl_3_–MeOH (2:1:1). Further purification by semi-preparative HPLC using a C18 column eluting with 90% of MeOH/H_2_O at a flow rate of 2.0 mL/min yielded compounds **1** (5.0 mg) and **2** (18.0 mg).

(±)-Pestalachloride D (**1**): colorless crystals; mp 225–227 °C; [α]^25^_D_ = 0 (*c* 1.00, CHCl_3_); UV λ_max_ (MeOH) nm: 209 (3.53), 283 (3.26), 327 (3.08) nm; IR (KBr) ν_max_ 3444, 1647, 1558, 1450, 1379, 1271, 1149 cm^−1^; ^1^H NMR and ^13^C NMR, see Table 1; EIMS *m/z* 422 [M]^•+^; HREIMS *m*/*z* 422.0678 [M]^•+^ (calcd for C_21_H_20_ Cl_2_O_5_, 422.0682).

X-ray Crystallographic Analysis of **1** and **2**. Colorless crystals of **1** and **2** were both obtained from MeOH. The crystal data were recorded at 293 K on an Agilent Gemini ultra diffractometer with Cu Kα radiation (λ = 1.54718 Å) and Mo Kα radiation (λ = 0.71073 Å). Their structures were solved by direct methods (SHELXS-97) and refined using full-matrix least-squares difference Fourier techniques. All non-hydrogen atoms were refined anisotropically, and all hydrogen atoms were placed in idealized positions and refined as riding atoms with the relative isotropic parameters. The crystallographic data for **1** and **2** have been deposited at the Cambridge Crystallographic Data Centre with the deposition numbers 896,051 and 896,954, respectively. Copies of the data can be obtained, free of charge, on application to the Director, CCDC, 12 Union Road, Cambridge CB21EZ, UK (Fax: +44(0)-1233-336033 or E-Mail: deposit@ccdc.cam.ac.uk).

Crystal data for **1**: C_21_H_20_Cl_2_O_5_, Mr = 423.27, monoclinic, space group *P*2_1_/c with *a* = 18.4179(4) Å, *b* = 9.2808(2) Å, *c* = 11.3163(3) Å, α = γ = 90°, β = 91.466(2)°, *V* = 1933.69(8) Å^3^, *Z* = 4, *Dx* = 1.454 mg/m^3^, μ(Cu Kα) = 3.289 mm^−1^, and *F*(000) = 880. Crystal dimensions: 0.42 × 0.41 × 0.31 mm^3^. Independent reflections: 2765 (*R*_int_ = 0.0334). The final *R*_1_ values were 0.0341, wR2 = 0.0891 (*I* > 2σ(*I*)). 

Crystal data for **2**: C_22_H_24_Cl_2_O_6_, Mr = 455.31, Triclinic, space group *P*-1 with *a* = 9.0363(18) Å, *b* = 10.083(2) Å, *c* = 12.356(2) Å, α = 68.534(3)°, β = 82.589(3)°, γ = 87.258(3), *V* = 1039.0(4) Å^3^, *Z* = 2, *Dx* = 1.455 mg/m^3^, μ(Mo Kα) = 0.350 mm^−1^, and *F*(000) = 476. Crystal dimensions: 0.43 × 0.31 × 0.17 mm^3^. Independent reflections: 3984 (*R*_int_ = 0.0299). The final *R*_1_ values were 0.0476, wR2 = 0.1329 (*I* > 2σ(*I*)). 

### 3.5.Biological Assays

The antibacterial activities against four bacterial strains, Gram-positive *M. tetragenus* (ATCC 13623), Gram-negative *E. coli* (ATCC 25922), *V. anguillarum* (ATCC 19019) and *V. parahaemolyticus* (ATCC 17802) were determined by a serial dilution technique using 96-well microtiter plates [14]. The compounds were dissolved in DMSO to give a stock solution. Bacterial species were cultured overnight at 37 °C in LB broth and diluted to 10^6^ cfu/mL when used. LB broth was used as a blank control, and DMSO was used as a negative control, while ciprofloxacin was used as a positive control. The plates were incubated at 37 °C for 24 h. The results were observed with a Multiskan Mk3 (Thermo Labsystems) at 630 nm. The zebrafish embryo teratogenicity assays were evaluated according to the described methods [4,5]. The agrochemical assays were evaluated by the methods described previously [15].

## 4. Conclusions

In summary, one new chlorinated benzophenone derivative, (±)-pestalachloride D (**1**), and its epimer (±)-pestalachloride C (**2**), have been isolated from a soft coral-derived fungus *Pestalotiopsis* sp. The structure of the new metabolite (**1**) was identified by comprehensive spectroscopic data. Both chiral HPLC analysis and single-crystal X-ray data indicated that **1** is a racemic mixture. Compound **2** exhibited toxicity toward zebrafish embryo, while **1** was completely inactive, and this is potentially due to the configurational differences observed in their crystal structures. This work will help to further understand the potential effect mechanisms of the zebrafish embryo teratogenicity assays. Furthermore, the separation of the enantiomers and further biological evaluation of the optically pure compounds are still in progress.

## Supplementary Files

Supplementary File 1 Supplementary Information (PDF, 472 KB)
