# Supplementary material for: (±)-Pestalachloride D, an Antibacterial Racemate of Chlorinated Benzophenone Derivative from a Soft Coral-Derived Fungus Pestalotiopsis sp
Source: Mar Drugs. 2013 Mar 28;11(4):1050–60. doi: 10.3390/md11041050 (PMC3705387; doi:10.3390/md11041050)
Supplement: Supplementary File 1 — Supplementary Information (PDF, 472 KB) [file marinedrugs-11-01050-s001.pdf]

Supplementary Information

Table of Content

Figure S1. ^{1}H NMR (400 MHz) spectrum of (±)-pestalachloride D (**1**) in $CDCl_3$. 2
Figure S2. ^{13}C NMR and DEPT (100 MHz) spectra of (±)-pestalachloride D (**1**) in $CDCl_3$. 3
Figure S3. EIMS spectrum of (±)-pestalachloride D (**1**). 4
Figure S4. HREIMS spectrum of (±)-pestalachloride D (**1**). 5
Figure S5. HPLC profiles of chiral Crownpak CR (+) column analysis of (±)-pestalachloride D (**1**). 6
Figure S6. ^{1}H NMR (400 MHz) spectrum of pestalachloride C (**2**) in $CDCl_3$. 6
Figure S7. ^{13}C NMR and DEPT (100 MHz) spectra of pestalachloride C (**2**) in $CDCl_3$. 7
Table S1. ^{1}H and ^{13}C NMR Data for (±)-pestalachloride D (**1**) and pestalachloride C (**2**). 8

Figure S1. ^{1}H NMR (400 MHz) spectrum of (±)-pestalachloride D (**1**) in $CDCl_3$.

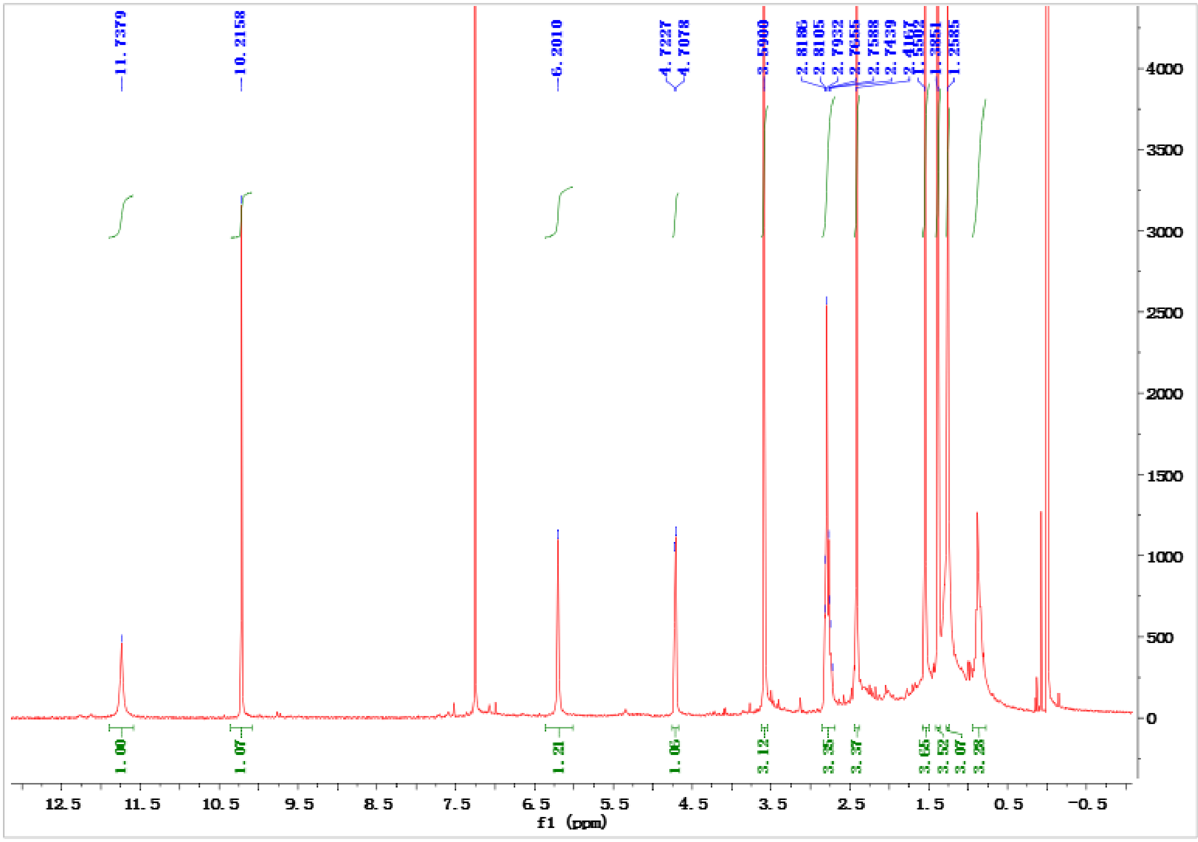

Figure S2. ^{13}C NMR and DEPT (100 MHz) spectra of (±)-pestalachloride D (**1**) in $CDCl_3$.

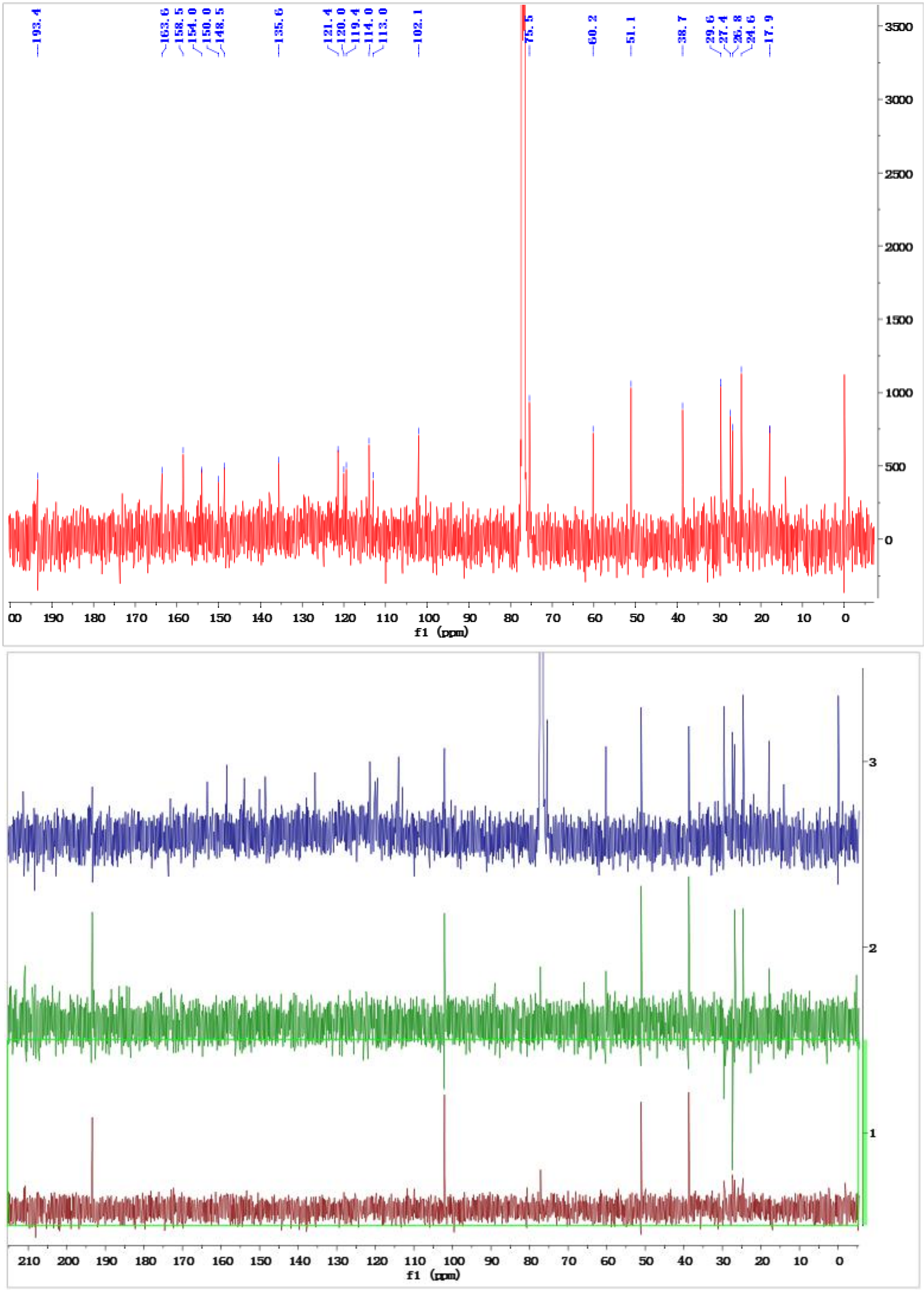

Figure S3. EIMS spectrum of (±)-pestalachloride D (**1**).

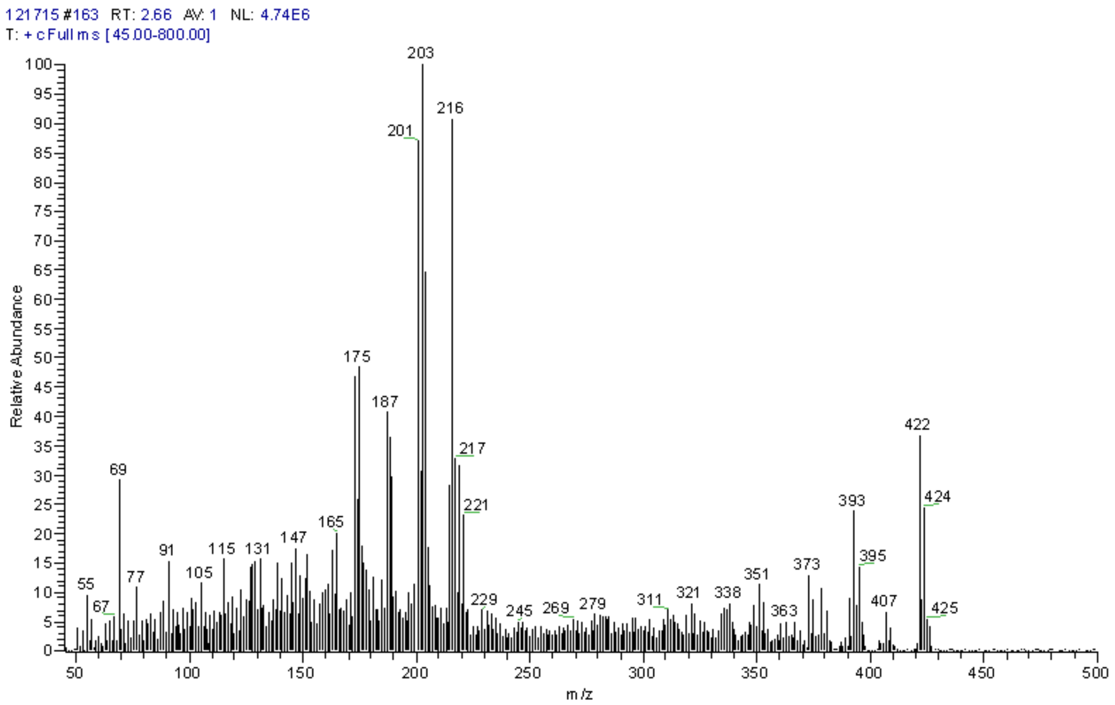

Figure S4. HREIMS spectrum of (±)-pestalachloride D (**1**).

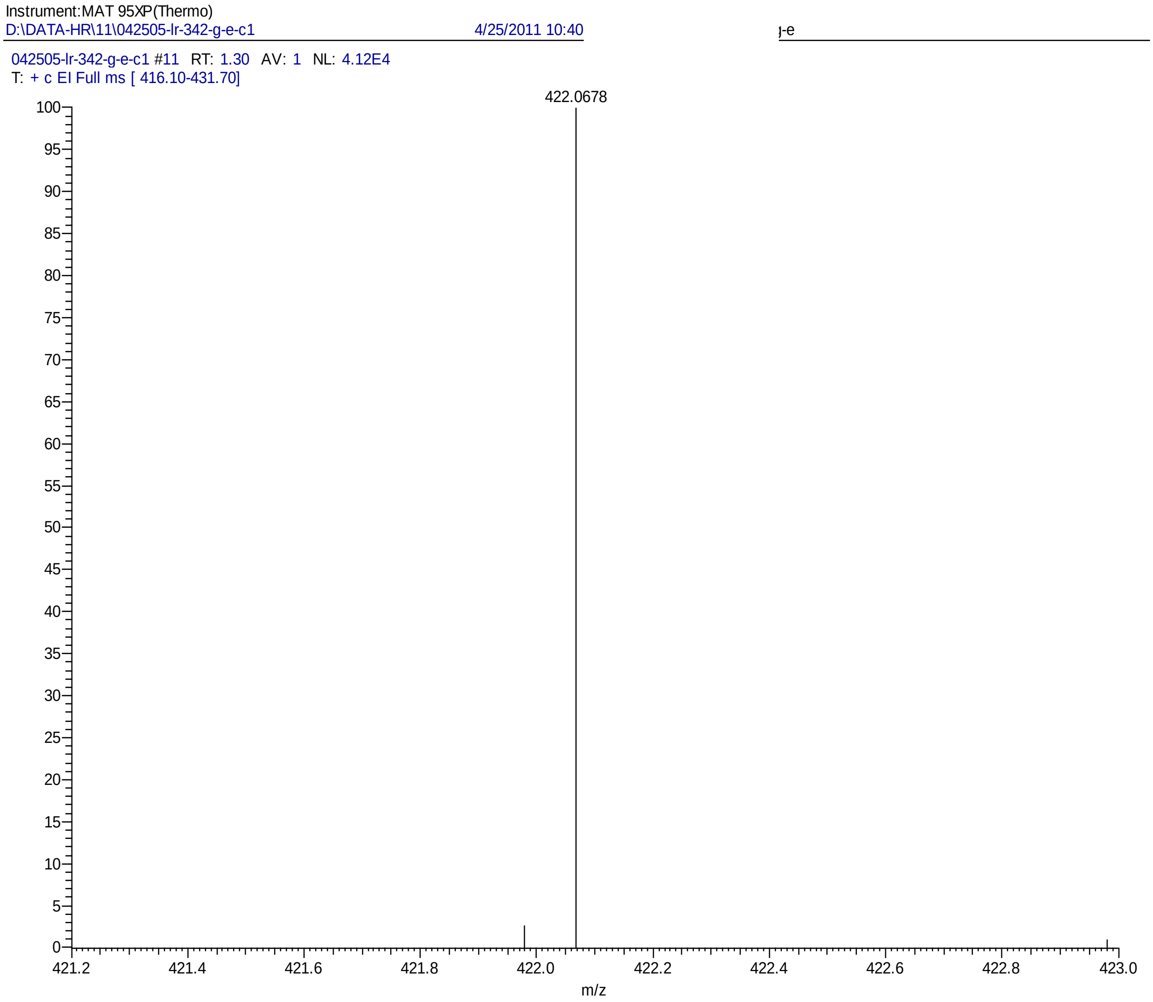

Figure S5. HPLC profiles of chiral Crownpak CR (+) column analysis of (±)-pestalachloride D (**1**).

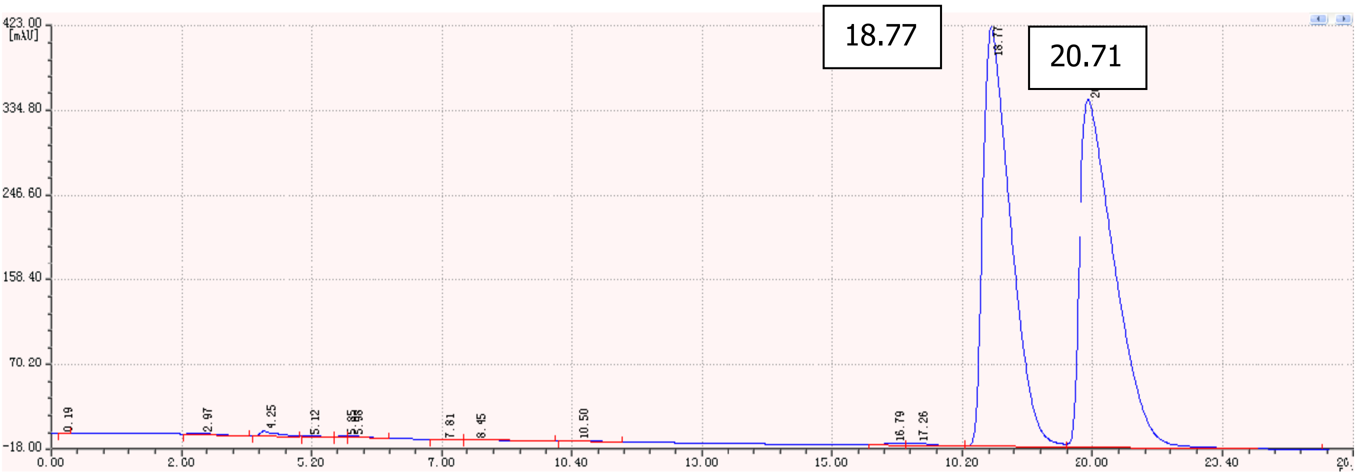

Eluted with 5% isopropanol in *n*-hexane at the flow rate of 0.8 mL/min.

Figure S6. ^{1}H NMR (400 MHz) spectrum of pestalachloride C (**2**) in $CDCl_3$.

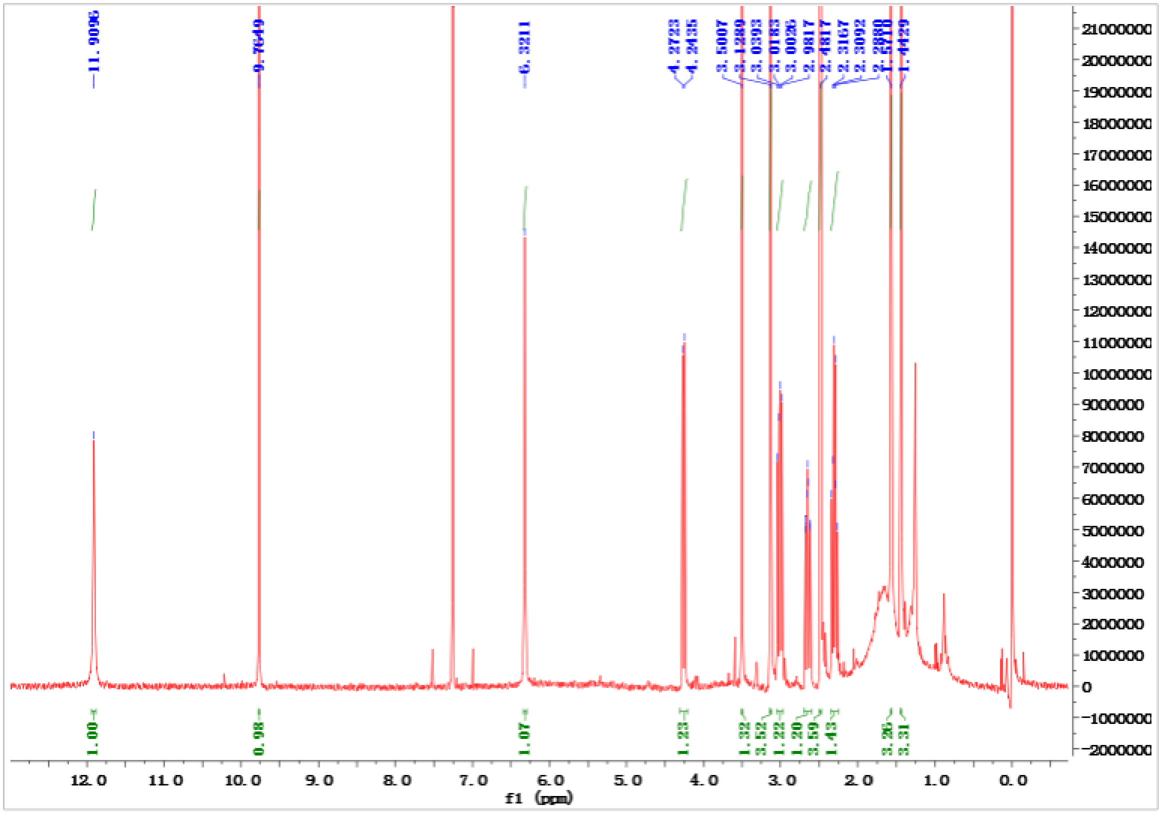

Figure S7. ^{13}C NMR and DEPT (100 MHz) spectra of pestalachloride C (**2**) in $CDCl_3$.

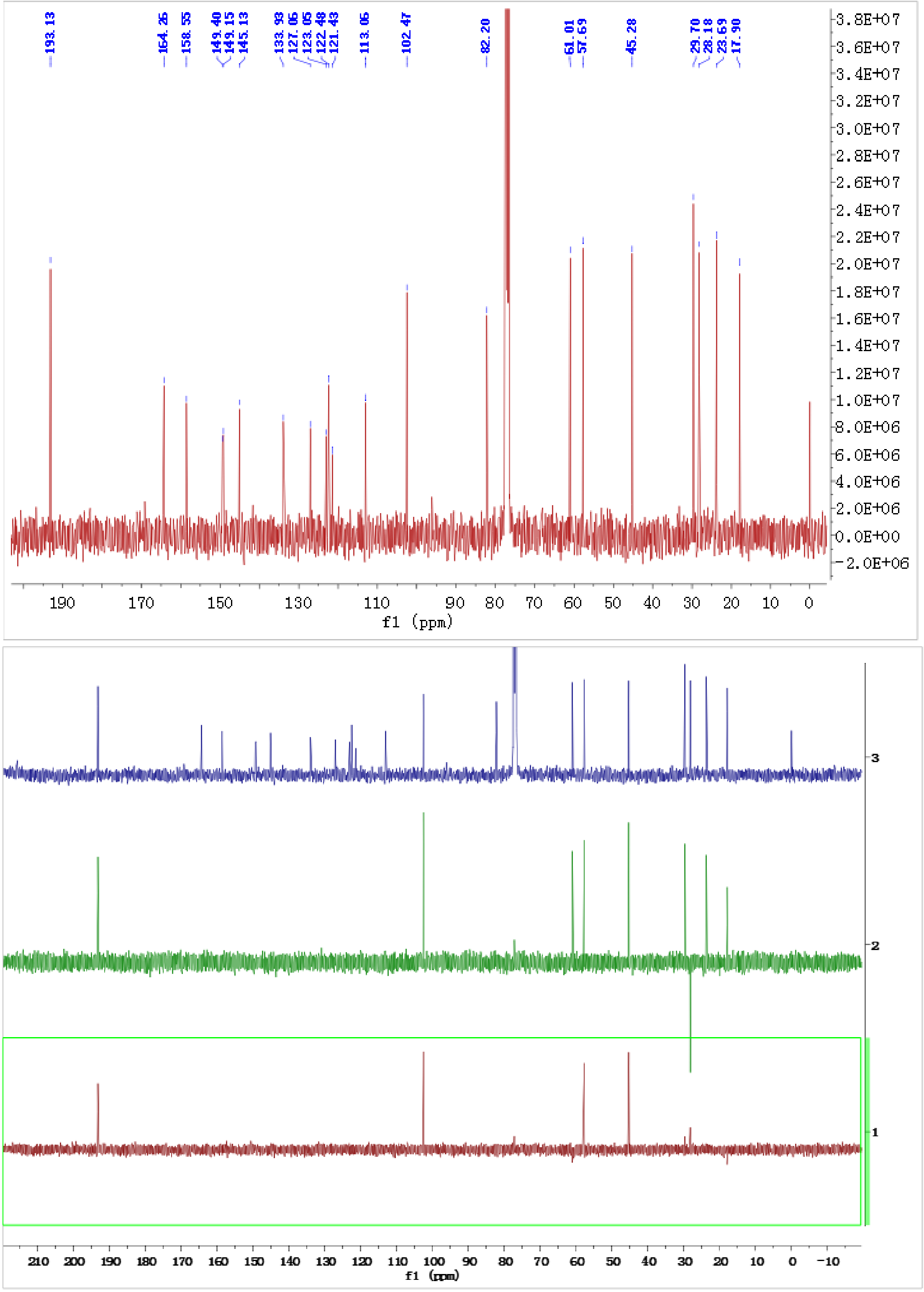

Table S1. ^{1}H and ^{13}C NMR Data for (±)-pestalachloride D (**1**) and pestalachloride C (**2**).

Position	**1** [a]		**2** [a]	
	δ_C	δ_H (*J* in Hz)	δ_C	δ_H (*J* in Hz)
1	193.4	10.21, s	193.1	9.76, s
2	112.9	—	113.1	—
3	163.6	—	164.3	—
4	102.1	6.20 (1H, s)	102.5	6.32 (1H, s)
5	158.5	—	158.6	—
6	120.0	—	127.0	—
7	148.5	—	145.1	—
8	38.7	4.71 (1H, d, 6.0)	45.3	4.26 (1H, d, 11.5)
9	121.4	—	123.1	—
10	150.0	—	149.4	—
11	114.0	—	121.4	—
12	135.6	—	133.9	—
13	119.4	—	122.5	—
14	154.0	—	149.2	—
15	17.9	2.42 (3H, s)	17.9	2.48 (3H, s)
16	60.2	3.59 (3H, s)	61.0	3.13 (3H, s)
1′ a	27.5	2.81 (2H, overlapped)	28.2	3.01 (1H, dd, 14.6, 8.4)
1′ b		—	—	2.64 (1H, ddd, 14.6, 11.3, 1.4)
2′	51.1	2.81 (1H, overlapped)	57.7	2.30 (1H, ddd, 11.5, 11.3, 8.4)
3′	75.6	—	82.2	—
4′	26.8	1.55 (3H, s)	29,7	1.57 (3H, s)
5′	24.6	1.38 (3H, s)	23.7	1.44 (3H, s)
3-OH		11.74, brs,		11.91,brs

[a] Measured at 400 MHz (^{1}H) and 100 MHz (^{13}C), $CDCl_3$.
